# Long-term immune response to SARS-CoV-2 infection and vaccination in children and adolescents

**DOI:** 10.1038/s41390-023-02857-y

**Published:** 2023-10-24

**Authors:** Sarah E. Messiah, Yashar Talebi, Michael D. Swartz, Rachit Sabharwal, Haoting Han, Emma Bergqvist, Harold W. Kohl, Melissa Valerio-Shewmaker, Stacia M. DeSantis, Ashraf Yaseen, Steven H. Kelder, Jessica Ross, Lindsay N. Padilla, Michael O. Gonzalez, Leqing Wu, David Lakey, Jennifer A. Shuford, Stephen J. Pont, Eric Boerwinkle

**Affiliations:** 1 Department of Epidemiology, Human Genetics and Environmental Sciences, The University of Texas Health (UTHealth) Science Center at Houston, School of Public Health in Dallas, Dallas, TX USA; 2 Center for Pediatric Population Health, UTHealth School of Public Health, Dallas, TX USA; 3 Department of Pediatrics, McGovern Medical School, Houston, TX USA; 4 Department of Biostatistics and Data Science, UTHealth Science Center at Houston, School of Public Health in Houston, Houston, TX USA; 5 Department of Epidemiology, Human Genetics and Environmental Sciences, UTHealth Science Center at Houston, School of Public Health in Austin, Austin, TX USA; 6 Department of Kinesiology and Health Education, University of Texas at Austin, Austin, TX USA; 7 Department of Health Promotion and Behavioral Sciences, The University of Texas Health Science Center at Houston, School of Public Health in Brownville, Brownsville, TX USA; 8 Department of Epidemiology, Human Genetics and Environmental Sciences, The University of Texas Health Science Center at Houston, School of Public Health, Houston, TX USA; 9 University of Texas System, Austin, TX USA; 10 The University of Texas Health Science Center Tyler, Tyler, TX USA; 11 Texas Department of State Health Services, Austin, TX USA

## Abstract

**Background:**

This analysis examined the durability of antibodies present after SARS-CoV-2 infection and vaccination in children and adolescents.

**Methods:**

Data were collected over 4 time points between October 2020-November 2022 as part of a prospective population-based cohort aged 5-to-19 years (*N* = 810). Results of the (1) Roche Elecsys® Anti-SARS-CoV-2 Immunoassay for detection of antibodies to the SARS-CoV-2 nucleocapsid protein (Roche *N*-test); and (2) qualitative and semi-quantitative detection of antibodies to the SARS CoV-2 spike protein receptor binding domain (Roche *S*-test); and (3) self-reported antigen/PCR COVID-19 test results, vaccination and symptom status were analyzed.

**Results:**

N antibody levels reached a median of 84.10 U/ml (IQR: 20.2, 157.7) cutoff index (COI) ~ 6 months post-infection and increased slightly to a median of 85.25 (IQR: 28.0, 143.0) COI at 12 months post-infection. Peak S antibody levels were reached at a median of 2500 U/mL ~6 months post-vaccination and remained for ~12 months (mean 11.6 months, SD 1.20).

**Conclusions:**

This analysis provides evidence of robust durability of nucleocapsid and spike antibodies in a large pediatric sample up to 12 months post-infection/vaccination. This information can inform pediatric SARS-CoV-2 vaccination schedules.

**Impact:**

- This study provided evidence of robust durability of both nucleocapsid and spike antibodies in a large pediatric sample up to 12 months after infection.
- Little is known about the long-term durability of natural and vaccine-induced SARS-CoV-2 antibodies in the pediatric population. Here, we determined the durability of anti–SARS-CoV-2 spike (S-test) and nucleocapsid protein (N-test) in children/adolescents after SARS-CoV-2 infection and/or vaccination lasts at least up to 12 months.
- This information can inform future SARS-CoV-2 vaccination schedules in this age group.

## Introduction

The ongoing SARS-CoV-2 pandemic continues to impact adults and children worldwide with new outbreaks reported in the Winter of 2023 due to the XBB.1.5 and Summer of 2023 due to the BA.2.86 Omicron subvariants.^1^ Through May 2023 over 15.5 million United States children and adolescents have been confirmed as COVID-19 positive.^2^ SARS-CoV-2 vaccinations became available for children in the spring of 2021 and continue to be one of the most effective public health tools to prevent severe, long or persistent COVID-19 illness. Yet, we know surprisingly little about the durability of antibodies acquired through infection or vaccine-induced SARS-CoV-2 antibodies in the pediatric population. While recent hospital-based studies have shown evidence pertaining to the durability of neutralizing antibodies in children up to 16 months after infection,^3^ much less information is available among children and adolescents who have not been hospitalized due to COVID-19 illness. This information is important in light of the continued emergence of new variants, vaccination schedule recommendations, and booster doses.

Among the limited studies in children, results show that antibody response may fluctuate by SARS-CoV-2 variant, vaccination status and age. One study has shown neutralizing antibody activity and breadth are similar in adults and children 4-months post-SARS-CoV-2 infection, and this persists for up to 12 months.^4^ Other studies have shown that despite naturally acquired antibodies that last up to a year, the Omicron variant poses particular challenges to protection, but that vaccination improved this response.^5,6^ Moreover, children who have experienced acute SARS-CoV-2 infection have significantly higher antibody levels that persist for longer periods of time compared to adults.^4,7,8^ Finally, one study of children showed that during the acute phase of SARS-CoV-2 infection (<1 month), children younger than 5 years of age had the highest levels of neutralizing antibodies compared to older age that remained at robust levels for up to 16 months.^3^

The investigative protocol for COVID-19 infection, which is population-based and age-stratified as defined by the World Health Organization (WHO),^9^ has two primary aims: (1) to gauge the prevalence of COVID-19 antibodies within the general population, providing insights into cumulative immunity, and (2) to assess the proportion of asymptomatic, pre-symptomatic, or subclinical infections, stratified by key demographics. In accordance with these guidelines, the objective of this study was to determine the duration of SARS-CoV-2 infection- and vaccine-induced antibody protection, and to identify factors associated with a decline in protection among a population-based cohort of children and adolescents aged 5 to 19 years. The working hypothesis posited that naturally acquired SARS-CoV-2 antibodies would persist for a minimum of 12 months, regardless of specific characteristics among pediatric participants.

## Methods

### Study design

Texas CARES (Coronavirus Antibody REsponse Survey) includes participants aged 5 to 90 years from the general population. The study details and methods have been described in detail elsewhere.^10^ Participants in the Texas CARES study received up to four free SARS-CoV-2 antibody tests from October 2020 to October 2022. The study is a partnership between the University of Texas Health Science Center at Houston School of Public Health, the Texas Department of State Health Services (DSHS), the University of Texas System, and Clinical Pathology Laboratories (CPL), a laboratory facility with over 200 statewide sites. All protocols were reviewed and approved by the University of Texas Health Science Center’s Committee for the Protection of Human Subjects and deemed public health practice by the Texas Department of State Health Services’ Institutional Review Board.

### Study recruitment

Texas CARES commenced enrollment in October 2020 across the state of Texas. Families of potential pediatric participants were informed about the survey through a variety of channels, including their healthcare providers, insurance carriers (for Medicaid-insured participants), media (radio, billboards), social media, school nurses and teachers, community events, and word-of-mouth. All information was delivered in both English and Spanish. This recruitment strategy was designed to reach a diverse range of potential participants, including those who may not have access to traditional healthcare settings or who may not be aware of other COVID-19 research studies. The study team also worked with community partners to ensure that the survey was accessible to families from all backgrounds.

### Study procedures

Parents or designated caregivers provided proxy informed consent for children and adolescents to participate in the Texas CARES study. Adolescents over the age of 12 had the option to sign assent and complete the questionnaire. No adolescents refused to provide assent or participate. Participants who consented to enroll in Texas CARES first completed a short online questionnaire to collect demographic information, employment status, baseline medical conditions and comorbidities, prior COVID-19 tests and diagnoses, physician diagnosis of COVID-19 and other chronic illnesses, and previous COVID-19 symptoms and severity. Once the participant completed the survey, orders were generated to take to a CPL facility of their choice to complete the antibody status blood draw. Participants typically received their results within 48 h. The study team took steps to ensure that the study procedures were safe and convenient for participants and their families. For example, participants were able to choose a laboratory facility that was convenient for them, and they typically received their results within a day or two. The study team also provided participants with information in English and Spanish about the study and their antibody test results.

### Study measures

#### SARS-CoV-2 antibody assay Roche diagnostics

SARS-CoV-2 antibody status was assessed using the Roche Elecsys® Anti-SARS-CoV-2 Immunoassay (Roche *N*-test). This assay detects high-affinity antibodies to SARS-CoV-2 using a modified recombinant protein representing the nucleocapsid (N) antigen.^10^ The assay is highly sensitive and specific, with a published sensitivity of 99.50% and a specificity of 99.8%.^11,12^

Several months after the start of Texas CARES, an immunoassay for the detection of antibodies to the SARS-CoV-2 spike protein (Roche *S*-test) became available. The Roche *S*-test was added to the study protocol to allow for monitoring of the combined impact of prior infection and the COVID-19 vaccine. Both the Roche *N*-test and the Roche *S*-test use whole blood and have a sensitivity and specificity exceeding 97%.

#### Electronic Questionnaire

Texas CARES uses an online questionnaire programmed in REDCap.^13,14^ The questionnaire was designed to be completed in 10–15 min by parents or designated caregivers as proxy respondents for children and adolescents. To increase the validity and reproducibility of the questionnaire, most of the questions and response formats were replicated from the COVID-19 PhenX Toolkit^15^ and the Behavioral Risk Factor Surveillance System^16^ questionnaires. The US Census race/ethnicity questions^17^ were also replicated. The questionnaire was seamlessly integrated with the informed consent process to ease the burden on respondents and maximize survey completion.

#### Weight groups

Body weight group was determined using the calculated body mass index (BMI) based on Centers for Disease Control and Prevention (CDC) age- and sex-adjusted BMI standardized values^18,19^ as follows: underweight (<5th percentile), healthy weight (≥5th to <85th percentile), overweight (≥85th to <95th percentile), and obesity (≥95th percentile).

#### Infection, hospitalization, and vaccine status

Infection status was defined as self-reported diagnosis by a doctor, positive PCR test, or positive N-antibody test after symptoms and close contact with a positive case. A total of 155 self-reported SARS-CoV-2 infections were removed from the analysis because they could not be confirmed with a positive N-antibody test, suggesting that these infections may have been caused by other viruses. Hospitalization status was defined by self-reported hospitalization for COVID-19. Vaccine status was defined by self-reported vaccination status at each of the four timepoints.

### Statistical analysis

Standard descriptive statistics (age, sex, race, ethnicity, residential density area, body mass index group, serostatus, and number of SARS-CoV-2 infections) were used to summarize the characteristics of pediatric participants. Continuous variables were presented as means and standard deviations, and categorical variables were presented as frequencies and percentages. Repeated-measures analysis allowed for within-individual comparisons across the four surveys.

To model symptomatic status, the analysis was limited to participants who reported at least one infection. Each infection was classified as symptomatic or asymptomatic based on self-reported symptom variables. The same generalized linear mixed-effects modeling strategy used for repeated measures above was applied, with symptomatic status as the outcome. The same variable selection strategy was applied, with the addition of vaccination status. Computations were implemented in R using the “lme4” package.

## Results

The final analytical dataset consisted of 810 children with complete data for four N-antibody tests and 734 children with full data available for four S-antibody tests. The mean age was ~12.3 years (SD 3.4) for each group, majority ages 10–14 years, female, non-Hispanic white, and with normal body mass index (Table 1).

**Table 1:** Descriptive characteristics, Texas CARES Pediatric Population who have completed 4 consecutive SARS-CoV-2 N and S antibody tests 2–3 months apart.

	*N* test (*n* = 810)^a^	*S* test (*n* = 734)^b^
Age (years), mean (SD)	12.39 (3.44)	12.34 (3.49)
Age group, years		
5–9, *n* (%)	176 (21.73%)	165 (22.48%)
10–14, *n* (%)	396 (48.89%)	350 (47.68%)
15–19, *n* (%)	238 (29.38%)	219 (29.84%)
Sex		
Female, *n* (%)	417 (51.55%)	375 (51.16%)
Male, *n* (%)	392 (48.45%)	358 (48.84%)
Ethnicity		
Non-Hispanic White, *n* (%)	532 (65.68%)	497 (67.71%)
Non-Hispanic Black, *n* (%)	10 (1.23%)	8 (1.09%)
Hispanic, *n* (%)	155 (19.14%)	126 (17.17%)
Asian, *n* (%)	0 (0.00%)	0 (0.00%)
Other, *n* (%)	113 (13.95%)	103 (14.03%)
Body Mass Index		
Underweight	33 (4.31%)	31 (4.48%)
Healthy Weight	515 (67.32%)	475 (68.64%)
Overweight	123 (16.08%)	104 (15.03%)
Obesity	94 (12.29%)	82 (11.85%)
COVID-19 Diagnosis, *n* (%)^c^	117 (14.44%)	101 (13.76%)
Chronic Disease, *n* (%)^c^	163 (23.76%)	144 (23.3%)
Vaccination Status^c^		
Full	523 (64.57%)	474 (64.58%)
Partial	10 (1.23%)	10 (1.36%)
None	277 (34.2%)	250 (34.06%)

^a^Chronic Disease Missing = 124; BMI Missing = 45; Sex Missing = 1.

^b^Chronic Disease Missing = 116; BMI Missing = 42; Sex Missing = 1.

^c^Reported at Time T4.

**Fig. 1:**
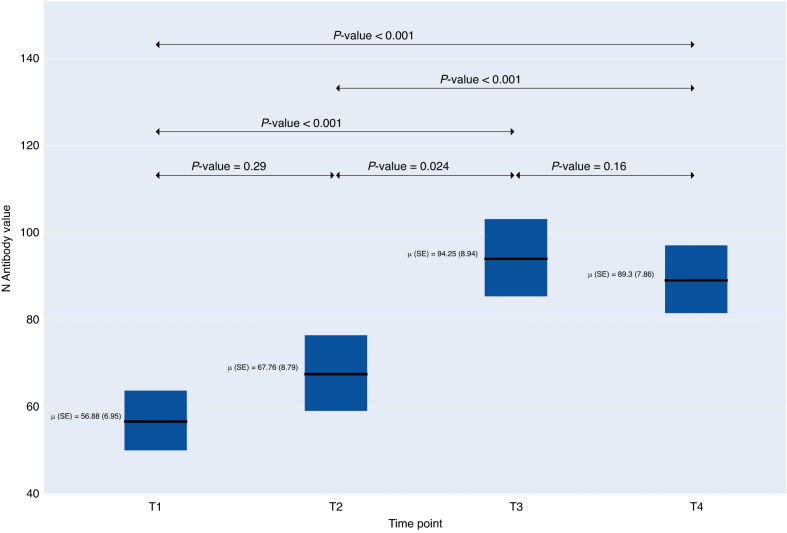
Durability of nucleocapsid (N) antibodies over time among children and adolescents participating in TX CARES. Nucleocapsid protein (Roche N-test) cut off index results over 4 time points 2020–2022 (*n* = 810). The symbol ‘μ’ represents the population mean.

Peak N-antibody levels were reached at a median of 84.1 U/ml (IQR: 20.2, 157.7) cutoff index (COI) approximately six months after time point 1 (baseline) (Fig. 1). Nucleocapsid (N) antibody levels increased slightly to a median of 85.25 U/ml (IQR: 28.0, 143.0) COI at 12 months after baseline. 98.1% of those with evidence of N antibodies at baseline assessment (32.8% of the sample) continued to have antibodies >12 months later (mean 12.29 months, SD 2.52). One child seroconverted from positive to negative status between their first and second antibody test, two children seroconverted from positive to negative status between their second and third antibody test, and two children seroconverted from positive to negative status between their third and fourth antibody test. Sixty-two children seroconverted from negative to positive between their first and second antibody test, 180 between their second and third test, and 147 between their third and fourth test, respectively.

The proportion of participants with positive N-antibody levels increased from 32.84% at the first time point to 79.51% at the fourth time point. Symptom status was not collected across all four time points (to reduce participant burden in T4) so no statistical tests were conducted to compare these two variables over time. There was no statistically significant difference in the presence of antibodies by sex, age group, or body mass index group (underweight, healthy weight, overweight, obesity) over the four-antibody measurement time points. There were significant differences over the four measurement time points in vaccination status, chronic disease status, COVID-19 disease status and repeat infections (*p* < 0.001 for all) (Table 2). The proportion of those with positive N antibodies among those with a chronic disease remained consistent across all four time points (21.69%, 22.70%, 21.75%, and 22.55%, respectively) as did those with no chronic disease (78.31%, 77.30%, 78.25%, 77.45%) (*p* < 0.001). Those who reported no COVID-19 disease and who had positive N antibodies increased from 32.33% at the first time point to 82.45% at the fourth time point while repeat infections were 8.65%, 0.92%, 8.93%, and 5.12% among those with positive N antibodies across the four time points, respectively. There were no statistical differences between symptom groups (symptomatic versus asymptomatic and mild/moderate versus severe) in N and S antibody levels.

**Table 2:** Sars-CoV-2 Nucleocapsid (N) antibody status over 4 timepoints (each separated by 2–3 months) by symptom status and severity and descriptive characteristics^a^.

	Timepoint 1 (*N* = 810)		Timepoint 2 (*N* = 810)		Timepoint 3 (*N* = 810)		Timepoint 4 (*N* = 810)		*p*-value^b^
	Positive	Negative	Positive	Negative	Positive	Negative	Positive	Negative	
	266 (32.84%)	544 (67.16%)	327 (40.37%)	483 (59.63%)	504 (62.22%)	306 (37.78%)	644 (79.51%)	166 (20.49%)	
Sex									
Males	126 (47.37%)	266 (48.99%)	153 (46.79%)	239 (49.59%)	239 (47.51%)	153 (50.0%)	309 (48.06%)	83 (50.0%)	0.79
Females	140 (52.63%)	277 (51.01%)	174 (53.21%)	243 (50.41%)	264 (52.49%)	153 (50.0%)	334 (51.94%)	83 (50.0%)	
Age group									
5–9 years	61 (22.93%)	115 (21.14%)	74 (22.63%)	102 (21.12%)	99 (19.64%)	77 (25.16%)	132 (20.5%)	44 (26.51%)	0.72
10–14 years	131 (49.25%)	265 (48.71%)	162 (49.54%)	234 (48.45%)	251 (49.8%)	145 (47.39%)	321 (49.84%)	75 (45.18%)	
15–19 years	74 (27.82%)	164 (30.15%)	91 (27.83%)	147 (30.43%)	154 (30.56%)	84 (27.45%)	191 (29.66%)	47 (28.31%)	
Body mass index group^c^									
Underweight	9 (3.67%)	24 (4.62%)	10 (3.28%)	23 (5.0%)	19 (3.98%)	14 (4.86%)	26 (4.26%)	7 (4.52%)	0.74
Healthy	164 (66.94%)	351 (67.5%)	204 (66.89%)	311 (67.61%)	317 (66.46%)	198 (68.75%)	406 (66.56%)	109 (70.32%)	
Overweight	40 (16.33%)	83 (15.96%)	52 (17.05%)	71 (15.43%)	81 (16.98%)	42 (14.58%)	103 (16.89%)	20 (12.9%)	
Obesity	32 (13.06%)	62 (11.92%)	39 (12.79%)	55 (11.96%)	60 (12.58%)	34 (11.81%)	75 (12.3%)	19 (12.26%)	
Vaccination status									
Full	28 (10.53%)	245 (45.04%)	70 (21.41%)	332 (68.74%)	238 (47.22%)	253 (82.68%)	369 (57.3%)	154 (92.77%)	< 0.001
Partial	3 (1.13%)	20 (3.68%)	18 (5.5%)	51 (10.56%)	14 (2.78%)	7 (2.29%)	9 (1.4%)	1 (0.6%)	
None	235 (88.35%)	279 (51.29%)	239 (73.09%)	100 (20.7%)	252 (50.0%)	46 (15.03%)	266 (41.3%)	11 (6.63%)	
Chronic disease status									
Yes	54 (21.69%)	132 (25.68%)	69 (22.7%)	117 (25.49%)	102 (21.75%)	84 (28.57%)	124 (22.55%)	39 (28.68%)	0.0810
No	195 (78.31%)	382 (74.32%)	235 (77.3%)	342 (74.51%)	367 (78.25%)	210 (71.43%)	426 (77.45%)	97 (71.32%)	
Symptom Status^d^									
Symptomatic	95 (55.23%)	21 (52.5%)	21 (80.77%)	2 (100.0%)	116 (89.23%)	9 (81.82%)	N/A	N/A	N/A
Asymptomatic	77 (44.77%)	19 (47.5%)	5 (19.23%)	0 (0%)	14 (10.77%)	2 (18.18%)	N/A	N/A	
COVID-19 Disease^e^									
Yes	180 (67.67%)	41 (7.54%)	34 (10.4%)	4 (0.83%)	167 (33.13%)	13 (4.25%)	113 (17.55%)	4 (2.41%)	< 0.001
No	86 (32.33%)	503 (92.46%)	293 (89.6%)	479 (99.17%)	337 (66.87%)	293 (95.75%)	531 (82.45%)	162 (97.59%)	
Hospitalization status due to COVID-19 disease									
Not Hospitalized	178 (98.89%)	40 (97.56%)	34 (100.0%)	4 (100.0%)	167 (100.0%)	13 (100.0%)	112 (99.12%)	4 (100.0%)	N/A
Hospitalized	2 (1.11%)	1 (2.44%)	0 (0%)	0 (0%)	0 (0%)	0 (0%)	1 (0.88%)	0 (0%)	
Repeat Infection^f^									
Yes	23 (8.65%)	3 (0.55%)	3 (0.92%)	1 (0.21%)	45 (8.93%)	1 (0.33%)	33 (5.12%)	0 (0%)	< 0.001
No	243 (91.35%)	541 (99.45%)	324 (99.08%)	482 (99.79%)	459 (91.07%)	305 (99.67%)	611 (94.88%)	166 (100.0%)	
Days Since T1									
Mean (SD)	0.0 (0.0)	0.0 (0.0)	98.4 (22.2)	96.7 (15.4)	196.9 (33.4)	197.4 (28.3)	368.6 (75.3)	369.3 (77.6)	N/A
	0.0 (0.0)		97.4 (18.4)		197.1 (31.5)		368.7 (75.7)		
*N* Value									
Mean (SD)	56.9 (57.6)	0.0 (0.1)	72.2 (74.3)	0.0 (0.1)	76.5 (71.4)	0.0 (0.1)	68.8 (62.8)	0.1 (0.2)	N/A
	18.7 (42.4)		29.2 (59.0)		47.6 (67.4)		54.7 (62.5)		

^a^N_missing_ sex = 1; N_missing_ body mass index = 45; N_missing_ age = 0.

^b^*p*-value calculated from likelihood ratio test of the simple model. *Hospitalization* and *symptom status* are considered proxy to the outcome and were not included in the model.

^c^based on standardized body mass index categories are as follows: (1) underweight = <5th percentile; (2) healthy weight = 5th–85th percentile; (3) overweight = 85th–<95th percentile and (4): obesity = >95th percentile.

^d^symptom status was not asked at timepoint 4, thus does not apply (N/A).

^e^self-reported positive diagnosis. Respondents could choose from the following 5 methods of how an infection was determined: (1) PCR test; (2) Home test; (3) Healthcare professional diagnosis with no test; (4) Presumed due to symptoms and close exposure to others with COVID-19; and (5) Other

^f^repeat infection means more than 1 reported infections before T1 or any infection between T1–T2, T2–T3, and T3–T4 if they have at least 1 infection before.

**Fig. 2:**
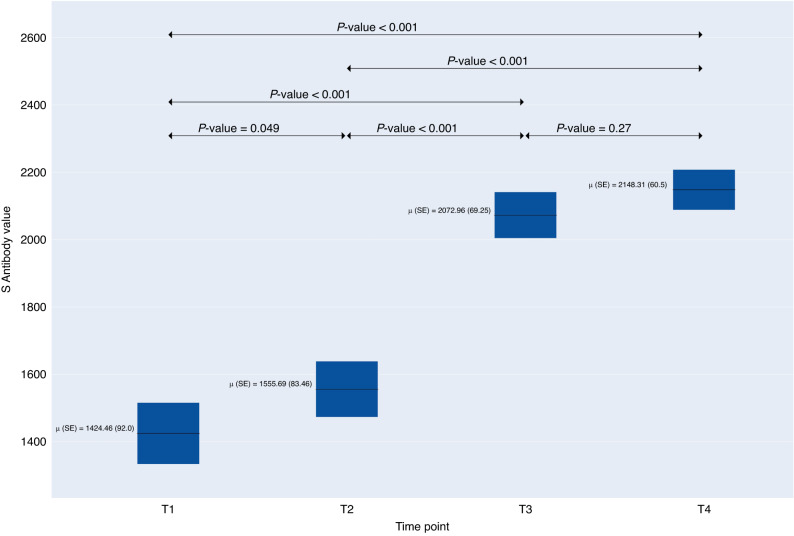
Durability of Spike (S) antibodies over time among children and adolescents participating in TX CARES. Spike protein (Roche S-test) cut off index results over 4 time points 2020–2022 (*n* = 734). The symbol ‘μ’ represents the population mean.

The Roche S antibody level is capped at 2500 U/mL (i.e., levels equal to or greater than 2500 U/mL are reported as ≥2500 U/mL). Peak spike protein (S) antibody levels were reached at a median of 2500 U/mL approximately six months after baseline and continued on at this level through the 4th test or approximately 12 months (mean 11.6 months, SD 1.20) (Fig. 2). 99.8% of those with S antibodies at baseline (68.8%) had antibodies >11 months later (mean 11.6 months, SD 1.19).

The proportion of participants positive for S-antibodies increased from 68.80% at the first time point to 98.91% at the fourth time point (*p* < 0.001). A smaller percentage of younger children aged 5-to-9 years had positive S antibodies versus 10-to-14-year-olds and 15-to-19-year-olds at all four time points (T1, T2, T3, T4 as denoted on tables) (*p* < 0.001). Not surprisingly, positive S antibody tests increased over the four time points among those fully vaccinated, but also increased in those with no vaccination (*p* < 0.001). Among those with positive S antibodies, the proportion of the sample reporting COVID-19 disease decreased from 37.43% at the first time point to 13.91% at the fourth time point while those reporting no disease peaked at time point two and decreased in the following third and fourth time points (62.57%, 94.83%, 74.89%, and 76.43%, respectively) (*p* < 0.001). There was no statistically significant difference in the presence of antibodies by sex, body mass index group, and chronic disease status. There were significant differences in age group, vaccination status and COVID-19 disease. Repeat infections were reported among 4.55% at timepoint 1, decreased to 0.61% at timepoint 2, then increased to 6.49% at timepoint 3 and slightly decreased to 4.13% at timepoint 4 among those with positive S antibodies, respectively (*p* = 0.005) (Table 3).

**Table 3:** Sars-CoV-2 spike (S) antibody status over 4 timepoints (each separated by 2–3 months) by individual and disease descriptive characteristics^a^.

	Timepoint 1 (*N* = 734)		Timepoint 2 (*N* = 734)		Timepoint 3 (*N* = 734)		Timepoint 4 (*N* = 734)		*p*-value^b^
	Positive	Negative	Positive	Negative	Positive	Negative	Positive	Negative	
	505 (68.8%)	229 (31.2%)	658 (89.65%)	76 (10.35%)	709 (96.59%)	25 (3.41%)	726 (98.91%)	8 (1.09%)	
Sex									
Males	233 (46.14%)	125 (54.82%)	315 (47.95%)	43 (56.58%)	345 (48.73%)	13 (52.0%)	353 (48.69%)	5 (62.5%)	0.059
Females	272 (53.86%)	103 (45.18%)	342 (52.05%)	33 (43.42%)	363 (51.27%)	12 (48.0%)	372 (51.31%)	3 (37.5%)	
Age group									
5–9 years	64 (12.67%)	101 (44.1%)	136 (20.67%)	29 (38.16%)	152 (21.44%)	13 (52.0%)	162 (22.31%)	3 (37.5%)	< 0.001
10–14 years	252 (49.9%)	98 (42.79%)	318 (48.33%)	32 (42.11%)	343 (48.38%)	7 (28.0%)	348 (47.93%)	2 (25.0%)	
15–19 years	189 (37.43%)	30 (13.1%)	204 (31.0%)	15 (19.74%)	214 (30.18%)	5 (20.0%)	216 (29.75%)	3 (37.5%)	
Body Mass Index Grouc^c^									
Underweight	21 (4.44%)	10 (4.57%)	28 (4.52%)	3 (4.11%)	30 (4.49%)	1 (4.17%)	30 (4.39%)	1 (12.5%)	0.26
Healthy	335 (70.82%)	140 (63.93%)	428 (69.14%)	47 (64.38%)	460 (68.86%)	15 (62.5%)	470 (68.71%)	5 (62.5%)	
Overweight	69 (14.59%)	35 (15.98%)	91 (14.7%)	13 (17.81%)	99 (14.82%)	5 (20.83%)	102 (14.91%)	2 (25.0%)	
Obesity	48 (10.15%)	34 (15.53%)	72 (11.63%)	10 (13.7%)	79 (11.83%)	3 (12.5%)	82 (11.99%)	0 (0%)	
Vaccination status									
Full	270 (53.47%)	3 (1.31%)	395 (60.03%)	1 (1.32%)	463 (65.3%)	0 (0%)	474 (65.29%)	0 (0%)	< 0.001
Partial	19 (3.76%)	4 (1.75%)	50 (7.6%)	10 (13.16%)	17 (2.4%)	0 (0%)	10 (1.38%)	0 (0%)	
None	216 (42.77%)	222 (96.94%)	213 (32.37%)	65 (85.53%)	229 (32.3%)	25 (100.0%)	242 (33.33%)	8 (100.0%)	
Chronic disease status									
Yes	115 (24.26%)	45 (20.83%)	145 (23.54%)	15 (20.27%)	159 (23.91%)	1 (4.0%)	144 (23.57%)	0 (0%)	0.40
No	359 (75.74%)	171 (79.17%)	471 (76.46%)	59 (79.73%)	506 (76.09%)	24 (96.0%)	467 (76.43%)	7 (100.0%)	
Symptom status^d^									
Symptomatic	104 (57.14%)	10 (62.5%)	21 (80.77%)	0 (0%)	123 (88.49%)	0 (0%)	0 (0%)	0 (0%)	N/A
Asymptomatic	78 (42.86%)	6 (37.5%)	5 (19.23%)	0 (0%)	16 (11.51%)	0 (0%)	0 (0%)	0 (0%)	
COVID-19 disease^e^									
Yes	189 (37.43%)	17 (7.42%)	34 (5.17%)	0 (0%)	178 (25.11%)	0 (0%)	101 (13.91%)	0 (0%)	< 0.001
No	316 (62.57%)	212 (92.58%)	624 (94.83%)	76 (100.0%)	531 (74.89%)	25 (100.0%)	625 (86.09%)	8 (100.0%)	
Covid-19 disease severity									
Not hospitalized	188 (99.47%)	16 (94.12%)	34 (100.0%)	0 (0%)	178 (100.0%)	0 (0%)	100 (99.01%)	0 (0%)	N/A
Hospitalized	1 (0.53%)	1 (5.88%)	0 (0%)	0 (0%)	0 (0%)	0 (0%)	1 (0.99%)	0 (0%)	
Repeat Infection^f^									
Yes	23 (4.55%)	1 (0.44%)	4 (0.61%)	0 (0%)	46 (6.49%)	0 (0%)	30 (4.13%)	0 (0%)	0.005
No	482 (95.45%)	228 (99.56%)	654 (99.39%)	76 (100.0%)	663 (93.51%)	25 (100.0%)	696 (95.87%)	8 (100.0%)	
Days Since T1									
Mean (SD)	0.0 (0.0)	0.0 (0.0)	95.7 (12.9)	95.5 (13.4)	194.5 (27.3)	197.0 (17.9)	347.9 (36.0)	352.6 (20.3)	N/A
	0.0 (0.0)		95.7 (12.9)		194.6 (27.0)		348.0 (35.9)		
*N* value									
Mean (SD)	1424.5 (1052.3)	0.4 (0.0)	1526.6 (992.8)	0.4 (0.0)	1954.6 (873.2)	0.4 (0.1)	2046.0 (810.4)	0.4 (0.1)	N/A
	980.2 (1094.2)		1368.6 (1048.8)		1888.0 (928.6)		2023.7 (833.5)		

^a^*N*_missing_ sex = 1; *N*_missing_ body mass index = 45; N_missing_ age = 0.

^b^*p*-value calculated from likelihood ratio test of the simple model. *Hospitalization* and *symptom status* are considered proxy to the outcome and were not included in the model.

^c^based on standardized body mass index categories are as follows: (1) underweight = <5th percentile; (2) healthy weight = 5th–85th percentile; (3) overweight = 85th–<95th percentile and (4): obesity = >95th percentile.

^d^symptom status was not asked at timepoint 4, thus does not apply (N/A).

^e^self-reported positive diagnosis. Respondents could choose from the following 5 methods of how an infection was determined: (1) PCR test; (2) Home test; (3) Healthcare professional diagnosis with no test; (4) Presumed due to symptoms and close exposure to others with COVID-19; and (5) Other

^f^repeat infection means more than 1 reported infections before T1 or any infection between T1-T2, T2-T3, and T3-T4 if they have at least 1 infection before.

## Discussion

The data reported here emphasize two practical implications. First, children have a robust response to previous COVID-19 infection and vaccination that lasts at least 12 months regardless of demographic characteristics. Second, it’s important to note that children generate antibodies in response to SARS-CoV-2 infection, irrespective of whether they experienced symptoms. These findings indicate that prior infection and/or antibodies induced by vaccination offer a certain level of protection against future infections, with this protection persisting for at least 1 year. To be specific, the results reveal that the overwhelming majority of children who had four consecutive antibody test results available for analysis over twelve months consistently retained SARS-CoV-2 N antibodies throughout the entire observation period and did not differ by age, sex, COVID-19 symptom status, severity of symptoms, and body mass index. These results suggest that infection-induced N antibodies persist and thus may provide some protection against future infection for at least 12 months. Results showed that differences between repeat infection groups (yes/no) was statistically significant at T1, T3, and T4 for N antibody levels. In other words, participants who had at least one repeated infection before their N antibody test had higher N antibody levels as expected. Similarly, spike antibodies lasted at least a year with no measurable decline over time. The difference between repeat infection groups (yes or no) was statistically significant at T3 only for S antibody levels. These results suggest that vaccination may offer protection against SARS-CoV-2 infection for at least 1 year. This evidence of robust durability of nucleocapsid and spike antibodies in a large pediatric sample up to 12 months post-infection/vaccination can inform pediatric SARS-CoV-2 vaccination schedules.

Studies have started to emerge in the literature reporting S and N antibody durability in pediatric samples with a combination of hospital and non-hospital/healthcare based.^3,20–22^ One important contribution to the literature of the findings here is the vast majority of participants in this sample were not hospitalized with COVID-19 illness. In fact, 44.77% of the sample was asymptomatic at the first data collection (survey) time point, even though they had positive N antibodies, suggesting high prevalence of asymptomatic SARS-CoV-2 infections in this population. Building on our previous analysis, 96% of Texas CARES children and adolescents with N antibodies at baseline retained them for over six months (mean 7.0 months, SD 0.97).^23^ No discernible variation in antibody presence was observed based on symptom status (asymptomatic vs. symptomatic), symptom severity (mild-moderate vs. severe), sex, age group, or body mass index category (underweight, healthy weight, overweight, obesity) across the three time points of antibody measurement that were assessed.^23^

In one of the largest cohort studies to date that included 548 children and 717 adults within 328 households, analysis showed that over two study time points over twelve months, the long-term humoral immune response to SARS-CoV-2 infection was longer duration in children than in adults, even after asymptomatic infection.^7^ In a second non-hospitalized cohort study that included 252 family clusters with COVID-19, results showed that anti–SARS-CoV-2 spike receptor-binding domain IgG persisted until 12 months after infection in both adults and children, with higher antibody peaks for younger participants at all follow-up time points.^4^ Their study design was very similar to Texas CARES and included three serial antibody data collection time points from April 1, 2020, to August 31, 2021. Our similar findings extend to October 2022 and both studies’ findings may help inform future COVID-19 vaccination scheduling strategies.

Other investigations of the longevity of S antibodies in adolescents found a significant decline in vaccinated adolescents over six months, but cross-reactivity against the Omicron RBD was observed after two vaccine doses.^24^ Furthermore, functional humoral activation against both the wild-type and Omicron variants of SARS-CoV-2 also exhibited a decline over time in vaccinated adolescent subjects. Conversely, a different study revealed that adolescents aged 11 to 16 years exhibited stronger binding antibody and neutralization responses against the spike protein of the wild-type SARS-CoV-2 virus, which is present in the Pfizer (BNT162b2) vaccine, one month after receiving the two-dose Pfizer vaccination. This response was more robust compared to adults aged 27 to 55 years.^25^ Our findings here show a robust response durability of S antibodies that not only increases in time but hits the test ceiling in terms of measurement amount by about six months and remains at this level for another two to three months. It should be noted that the S test detects spike protein that is a result of both the vaccination as well as naturally induced infection.

Results by time point four or mid-fall 2022 show that the majority of children and adolescents who are participating in Texas CARES have acquired antibodies through COVID-19 infection (79.51%) while almost the entire sample (98.91%) has evidence of protection via S antibodies. This is encouraging as new variants continue to emerge and quickly circulate. Based on self-reported data from individuals who underwent both antibody and antigen or PCR tests, it appears that SARS-CoV-2 has been extensively prevalent within the pediatric population. It remains crucial to maintain ongoing surveillance of COVID-19-related symptoms, particularly considering the widespread circulation of influenza and RSV among the pediatric population in the United States. Additionally, it’s essential to adhere to recommended vaccination schedules, which include booster shots, to optimize protection against future illnesses, especially among the youngest age cohorts.

Finally, it will also be important to monitor SARS-CoV-2 natural and vaccine-induced antibody protection in children beyond 12 months. The long-term effects of COVID-19 in children remain largely unknown, and understanding the duration of antibody protection can inform public health strategies.^26^ Additionally, emerging variants of the virus may pose new challenges, and tracking antibody levels can help assess the need for booster shots or modified vaccines in this vulnerable population.^27^ Previous studies have shown that children can be asymptomatic but infectious, affecting overall community transmission rates.^28^ Therefore, an extended period of observation is essential to ensure the continued safety and well-being of children and to effectively manage the ongoing COVID-19 pandemic.

### Study strengths and limitations

The findings presented herein should acknowledge specific limitations. Firstly, a limitation arising from the sampling frame is the presence of selection bias, meaning that parents who suspected their child or an adult in the household had contracted SARS-CoV-2 might have been more inclined to participate. Nonetheless, a significant number of children reported being asymptomatic. Secondly, it’s worth noting that the Roche Elecsys® Anti-SARS-CoV-2 Immunoassay can detect the presence of IgM, IgA, or IgG antibodies but cannot distinguish between these specific immunoglobulin antibody statuses individually. Thirdly, genomic sequencing was not conducted to genetically characterize pre-Delta and other Omicron variants. Fourth, no information was available on when actual infection occurred pre-baseline, and thus, our antibody durability estimates are as conservative as possible in that the baseline/time point 1 was considered the time of infection and/or vaccination for all analyses. Fifth, we did not collect detailed information on hospitalization status, so we do not know if a participant was admitted due to COVID-19 illness or the parent presented to a hospital for testing only. However, we have very few (<3) who stated their child was hospitalized due to COVID-19 illness. Finally, all COVID-19 disease and vaccination data were self-reported. Strengths of the study include that, to our knowledge, this is the only US pediatric sample in the literature with four consecutive antibody tests over at least 1 year with the addition of survey data collected on disease and symptom status over a 2-year period.

## Conclusions

Results here indicate that a significant proportion of non-hospitalized children, who were monitored for a duration of up to 12 months and had four consecutive antibody test results available for analysis, consistently maintained both SARS-CoV-2 N and S antibodies throughout the entire observation period. This remained true regardless of factors such as age, sex, COVID-19 symptom occurrence and severity, as well as body mass index. These findings suggest that antibodies generated from infection and vaccination persist and, as a result, could offer a certain degree of protection against future infections for at least 12 months. While the effectiveness of this response may fluctuate in the presence of different variants, the overarching trend of persistence remains, and this evidence can help inform vaccination schedules for pediatric SARS-CoV-2 vaccination efforts.

## Data Availability

Texas CARES investigators are committed to data sharing. Granular results and user-specified data summaries are currently publicly available on the Texas CARES portal (https://sph.uth.edu/projects/texascares/dashboard). When baseline recruitment is complete, a de-identified individual level dataset will be available for download from the same portal.
